# A scoping review of community-based post-opioid overdose intervention programs: implications of program structure and outcomes

**DOI:** 10.1186/s40352-022-00201-w

**Published:** 2023-01-28

**Authors:** Amelia Bailey, Calla Harrington, Elizabeth A. Evans

**Affiliations:** 1 Department of Health Promotion and Policy, School of Public Health and Health Sciences, University of Massachusetts Amherst, Amherst, MA 01003 USA; 2 Department of Behavioral and Social Sciences, School of Public Health, Brown University, Providence, RI 02912 USA

**Keywords:** Post overdose, Intervention, Opioid use disorder (OUD), Scoping review, Law enforcement, Deflection

## Abstract

**Background:**

An emergent intervention to address the opioid epidemic is the use of multidisciplinary outreach teams which connect an individual in the community to healthcare resources after the experience of an opioid overdose. While these interventions are receiving federal funding, less is known empirically to inform future interventions. Understanding the process and outcomes of these interventions is advisable due to the novel partnerships of public health and law enforcement agencies who sometimes hold divergent goals. The objective of the present review was to describe program structure and evaluated outcomes of community-based post-overdose interventions.

**Results:**

A search of PubMed, PsycInfo, and Web of Science yielded 5 peer-reviewed articles that detail the implementation and outcomes of interventions delivered in the United States published from 2001 to July 2021. Most interventions used a multidisciplinary outreach team and referenced first responder data to contact individuals who recently experienced an overdose at their residence. Services offered often included referral to substance use treatment, recovery coaches, and social services. Method of outreach, evaluation measures, and outcomes varied. From the available literature, facilitators of program engagement included communication, information sharing, and leadership buy-in among multidisciplinary partners.

**Conclusions:**

Future studies could benefit from exploration of service provision in rural areas, for family affected by overdose, and for minoritized populations. Community-based post-overdose interventions utilizing a law enforcement partnership are emergent with promising yet limited examples in empirical literature.

**Supplementary Information:**

The online version contains supplementary material available at 10.1186/s40352-022-00201-w.

## Background

### Public health need

In the United States, rates of non-fatal and fatal opioid overdoses have increased markedly since 2020 (Ciccarone et al., 2021). Estimates based on provisional data indicate an approximate 30% increase in drug overdose deaths from 2019 to 2020, primarily involving opioids (Ahmad et al., 2021). Additionally, compared to the general population, people with opioid use disorder (OUD) face increased health risks (Jones & McCance-Katz, 2019; Winkelman et al., 2018). Increased access to healthcare (i.e., medication for opioid use disorder, harm reduction) is one key strategy for addressing the toll of OUD on individuals and communities.

### Emergent interventions

The standard evidence-based treatment for OUD is medication to treat opioid use disorder (MOUD). MOUD is administered at hospitals, inpatient and outpatient programs, and other certified institutions in the United States (Krawczyk et al., 2021), but it remains underutilized (Hoffman et al., 2019). To address this gap over the past decade, evidence-based models have included MOUD in criminal justice settings (Simon, Rich, & Wakeman, 2021), hub and spoke models of healthcare delivery (Brooklyn & Sigmon, 2017), and telehealth expansion (Frank & Lin, 2022). Recent efforts have focused on implementation of novel programs to engage more people with OUD in evidence-based treatment (Khatri & Perrone, 2020; Sigmon, 2019). These emergent programs have included, for example, increased provision of MOUD (Reif et al. 2020) and naloxone (Clark et al., 2014; Moustaqim-Barrette et al., 2021), recovery support (Bassuk et al., 2016; Magidson et al., 2021), and post-overdose intervention programs.

### Post-overdose interventions

Post-overdose interventions aim to engage an individual who has recently experienced an overdose by connecting them with healthcare resources to reduce future overdose (Davoust et al., 2021). Post-overdose interventions are a “naloxone-plus” form of deflection, where individuals are referred to health and social services after administration of naloxone to decrease future public safety concerns (Charlier & Reichert, 2020; Charlier et al., 2022). Services include information on treatment options, harm reduction, recovery support, and/or other social services. Post-overdose interventions typically intervene within 72 hours or within 1 week to one month after an overdose (Davoust et al., 2021).

Many post-overdose interventions are based in hospitals where individuals receive medical care (Bagley et al., 2019). These programs do not encompass overdose events in which emergency medical services (EMS) or police are dispatched to the site of an overdose, but the individual declines transportation to a hospital (Bergstein et al., 2021; Wagner et al., 2019). An estimated 58–80% of emergency responder calls to an overdose result in transportation to a hospital (Harrison et al., 2021; Zozula et al., 2021). In effect, hospital-based post-overdose interventions exclude individuals who do not go to the hospital (Wagner et al., 2019), pointing to a gap in care for those who may be most in need, including women and vulnerable populations (Harrison et al., 2021; Zozula et al., 2021). The consequences of this gap in care may be extreme, as mortality risk is higher in the post-overdose period (Weiner et al., 2020).

To address this service gap, communities have organized to create community-based post-overdose interventions. The Substance Abuse and Mental Health Services Administration (SAMHSA) has prioritized funding for the planning, implementation, and delivery of post-overdose interventions to reduce overdose mortality in the US (Substance Abuse and Mental Health Services Administration, 2020). While community-based post-overdose interventions are implemented, the evidence base for these programs is still emerging and not yet fully understood (Bagley et al., 2019).

### Community-based post-overdose interventions

Two prior scoping reviews have synthesized the literature on community-based post-overdose intervention programs. Bagley et al. (2019) synthesized what is known about post opioid-overdose interventions in community (*n* = 16) and hospital settings (*n* = 11). Champagne-Langabeer et al. (2020) further explored 27 “out-of-hospital” post-overdose interventions with a focus on EMS-led interventions. Both reviews found post-overdose interventions addressed a gap in healthcare service and were led by multidisciplinary teams but noted variability in intervention design and limited monitoring of outcomes. Most information derived from Bagley et al. (2019) centers on gray literature (i.e., non-peer reviewed government reports, program websites). Gray literature has potential limitations in relation to study design, construct measurement, and data quality because it does not require the same oversight and regulations as peer-reviewed publications (Adams, Smart, & Huff, 2017).

Notably, few of the interventions in prior scoping reviews included programs that engaged law enforcement partners in program operation (Bagley et al., 2019; Champagne-Langabeer et al., 2020). Law enforcement are potential key partners in public health initiatives that aim to reduce harms of the opioid epidemic (Becker, 2021; Goulka, Del Pozo, & Beletsky, 2021). Law enforcement staff can refer individuals to treatment and other resources (Schiff et al., 2017; Yatsco et al., 2020a). At the same time, inclusion of law enforcement can present differing views of how to treat OUD (Saloner et al., 2018) and yield other complications. Though law enforcement is increasingly involved in post overdose outreach programs, there is also a growing movement to de-center or remove law enforcement from behavioral health interventions entirely. For example, some behavioral health programs (such as “CAHOOTS” in Oregon) purposefully enlist behavioral health workers to manage crisis calls that would often fall to law enforcement in other jurisdictions (Waters, 2021).

### Current review

To further grasp the context of post-overdose interventions, a scoping review methodology was selected to survey current peer-reviewed literature (for example, Finlay et al., 2019). In contrast to other review typologies, the scoping review methodology provides an overview of information on a limited, emergent concept. Scoping reviews do not seek to draw conclusions on efficacy or effectiveness but instead seek to clarify concepts (e.g., what is a community based post-overdose intervention) and identify gaps in research (i.e., what is unknown, what requires more evidence) (Daudt et al., 2013; Munn et al., 2018; Peters et al., 2015). This scoping review will address current knowledge on community-based post-overdose intervention programs including program structure, evaluation design, and reported outcomes. We specifically sought articles with law enforcement partnerships (i.e., programs that are partnered with law enforcement) to conduct outreach activities. The review will extrapolate data from peer-reviewed literature on the defined programs and provide guidance for implementation of interventions with similar staff capacity and workflow requirements.

## Methods

### Design

The scoping review was conducted using the PRISMA checklist modified for scoping reviews (Tricco et al., 2018) (Appendix A).

### Eligibility criteria

We searched for articles published between January 2001 and July 2021 to capture the most recent two decades of addiction research. Included articles detailed the following intervention activities:

1. Received notification of an individual’s opioid overdose within one month of the incident
2. Received notification of opioid overdoses that occurred in the community (i.e., at residence, on the street)
3. Contacted individual(s) who had experienced or witnessed an opioid overdose
4. Attempted to connect or successfully connected individual(s) with health resources in the community

Articles were included when process or outcome data was reported. Additionally, we used an additional key term to search for law enforcement to explicitly search for law enforcement-partnered interventions. To identify additional literature, we examined the reference lists of articles meeting the inclusion criteria, first by title and then by abstract.

Interventions based primarily in a hospital (i.e., emergency department), police station, or fire station were excluded. These programs were drastically different in design and needs than interventions taking place in the community. Articles were excluded if the intervention’s primary purpose was to deliver pre-overdose prevention services. Articles were excluded if not published in the English language or published on interventions outside of the United States. Scoping and systematic reviews were excluded. In particular, Formica and colleagues (Formica et al., 2018; Formica et al., 2021) offer valuable knowledge on post-overdose programs, which we consider further in relation to our results (see discussion). However, these review papers present a synthesis of information on more than one program. We excluded review papers because it was not possible to identify and link individual program activities to outcomes, precluding ability to understand whether and how intervention components operate.

### Information sources

We searched PubMed, PsycInfo, and Web of Science for peer-reviewed literature.

### Search

Search terms included post-overdose, intervention, opioid overdose, community, and law enforcement. Appendix B presents a full numbered list of search terms, including phrase variations.

### Selection of articles

**Fig. 1:**
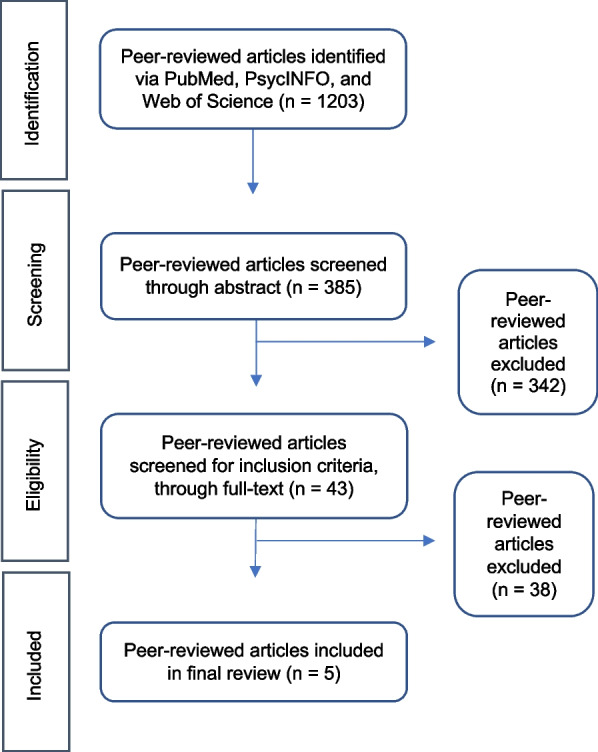
Selection of articles from peer-reviewed database search (PubMed, PsycInfo, Web of Science)

Figure 1 illustrates the number of articles found and reviewed for this process. From the search results, one reviewer examined the article’s title and abstract for inclusion. Abstracts that contained one or more of the intervention activities detailed in the eligibility criteria were included in the next round of review. We obtained full text articles for the second phase of review. Two reviewers independently completed a secondary screening by reading the full text of each article. After independent review of each article, the reviewers discussed article selection. Differing opinions occurred a few times and were resolved in light of the inclusion criteria and with further discussion. If the article did not meet the full inclusion criteria at this time, it was excluded. The data extraction process was completed by two reviewers.

### Synthesis of results

The articles included for final review were read through and coded for information corresponding to the relevant measures in Table 1. The measures were used to provide insight into program commonalities, differences, and salient outcomes.

**Table 1:** Community-based post-overdose interventions documented in empirical literature, 2001–2021

Reference	Program, Year	Key Partners	Participant Identification	Services Offered	Participants	Study Design and Results
Scott et al., 2020	Recovery Initiation and Management after Overdose (RIMO), Chicago, IL, Not Reported	Chicago FD EMS, Lighthouse Institute, research personnel, linkage managers. SAMHSA funding.	EMS data released to study team after naloxone administration and patient authorization. Participants not in treatment, use opioids, at least 18 years old. Linkage managers call participant. Second contact peer outreach workers to residence. Individual is transported to study site, screened, enrolled.	Intervention group: treatment referral (MOUD, detox, other), linkage managers check-in weekly and refer to social services. Control group: treatment options flyer.	*N* = 33 27.3% females; 66.7% Black, 15% Latinx, 9.1% White	Participants randomized into intervention (*n* = 17) or control (16). Mixed-methods: baseline survey, follow-up survey and focus group. Intervention group was significantly more likely to initiate treatment (81% vs. 35%), especially MOUD (81% vs. 18%), and to stay engaged in MOUD after 30-day period (44% vs. 6%). Intervention focus group (*n* = 9 from intervention) reported persistent and motivational follow-up aided engagement.
Langabeer et al., 2021	Houston Emergency Response Opioid Engagement System (HEROES), Houston, TX, 2018	University of Texas Health Science Center, Memorial Hermann Hospital, Houston FD, Houston Recovery Center. Health and Human Services Commission of Texas and SAMHSA funding.	EMS and ED data released to program after naloxone administration. Participants not in treatment, use opioids, at least 18 years old, valid address. Within 168-hours of event, peer recovery coach and paramedic conduct residential outreach to screen and enroll participant. Patient navigator schedules treatment appointments.	MOUD induction (buprenorphine) within 24-hours, referral to outpatient, weekly check-in and counseling. Referral to social services.	*N* = 70 Socio-demographic not reported.	Quantitative administrative case data. 1000 cases and 212 were available at first contact. Of those contacted, 70 enrolled in treatment (33%). Authors challenges to program success: cost of outreach and establishing health data agreement. Facilitators: outreach team share personal experience of substance use and risks.
Langabeer et al., 2020	Houston Emergency Response Opioid Engagement System (HEROES), Houston, TX, 2018	University of Texas Health Science Center, Memorial Hermann Hospital, Houston FD, Houston Recovery Center. SAMHSA funding.	EMS and ED data released to program after naloxone administration. Participants not currently in treatment, use opioids, at least 18 years old. Within 168-hours of event, peer recovery coach and paramedic conduct residential outreach to screen and enroll participant. Patient navigator schedules treatment appointments.	MOUD induction (buprenorphine) within 24-hours, referral to outpatient, weekly check-in and counseling. Referral to social services.	*N* = 34 55.9% male; 38.2 mean age; 61.8% White, 23.5% Black, 8.8% Latinx; 76.5% homeless; 75% unemployed; 79.4% no health insurance	Quantitative administrative case data. 251 cases, 103 contacted, and 34 (33%) were enrolled in study. Retention at 30-days was 88% (1 lost contact; 3 chose to stop); at 90-days retention was 56% (6, 9). No reported overdoses or deaths. Authors challenges to program success: inability to reach individuals and to access public health insurance. Facilitators: providing treatment and services to vulnerable population and strong agency relationships.
Yatsco et al., 2020	Houston Emergency Response Opioid Engagement System (HEROES), Houston, TX, 2018	University of Texas Health Science Center, Houston PD, psychiatric and recovery centers. Department of Justice Bureau of Justice Assistance funding.	Law enforcement data released to program after overdose or high-risk behavior. Participants able to enter treatment, use opioids, at least 18 years old. Within 168-hours of event, law enforcement conduct residential outreach to screen and enroll participant. Patient navigator schedules treatment appointment within 48-hours.	MOUD induction (buprenorphine) within 24-hours, referral to outpatient, weekly check-in and counseling. Referral to mental health and social services.	*N* = 24 75% male; 31.6 mean age; 87.5% White non-Latinx or Latinx	Quantitative administrative case data. Treatment engagement of 23% for those who were contacted. Authors shared challenges to program success: slow growth of program, participant distrust of law enforcement, and potential bias of which participants receive law enforcement treatment referral.
White et al., 2021	The Tempe First-Responder Opioid Recovery Project (ORP), Tempe, AZ, 2020	Tempe PD, Tempe FD, EMPACT (behavioral health), Arizona State University, California State University Long Beach. SAMHSA funding.	Police release 9–11 overdose data to program’s behavioral health counselor 24/7 hotline. Participant eligibility criteria not reported. Peer support specialist goes to individual’s location to collect contact information and discuss program. Within 24-hours, a navigator conducts follow-up to provide services for individual and family/friends.	Referrals include: naloxone, state-designated mental health treatment, substance use treatment (outpatient, residential), and social services. Navigator contact for 45 days.	*N* = 81 32.1 mean age; 69.8% White, 14.6% Black, 12.5% Latinx, 27% homeless	Mixed-methods: administrative data on cases and qualitative data from partner interviews. 81 individuals survived overdose. Of those contacted (63, 78%), 34 (54%) accepted navigation to services. Most often state-designated mental health (*n* = 16), outpatient (15), residential (7), and naloxone (67). Key partner facilitators for program success: collaboration and communication among partners, continuous meetings, understanding of agency norms, and multisectoral relationships. Anticipated benefits: officer commitment and tools to save lives.

Caption: Abbreviations: EMS = emergency medical services; ED = emergency department; FD = fire department; PD = police department; SAMHSA = Substance Abuse and Mental Health Services Administration; social services = housing, insurance, employment

## Results

From the search, 1203 articles were found. Of those, five articles fit the inclusion criteria for the current study. Four of the papers described implementation of programs (Langabeer et al., 2020; Langabeer et al., 2021; White et al., 2021; Yatsco et al., 2020b) and one described a research study (Scott et al., 2020). The five articles represent three unique programs: Houston Emergency Response Opioid Engagement System (HEROES), Recovery Initiation and Management after Overdose (RIMO); and Tempe First-Responder Opioid Recovery Project (Tempe First-Responder ORP).

### Program structure

The programs were located in three states (Texas, Arizona, Illinois), encompassing urban areas (highest population 2.7 million individuals) (United States Census Bureau, 2020). All programs were founded in or after 2018.

All programs had a team of key partners which steered operations. Programs’ key partners were comprised of both first responders (i.e., law enforcement or EMS) and public health personnel. Key partners typically represented police departments, fire departments, hospitals, and behavioral health organizations. Local universities often served as program evaluators.

Every program’s target population was a person who had experienced an overdose. All programs received data from police or EMS to identify people who had overdosed. All programs had relationships with police or EMS to either directly call the program staff or directly enter 9–11 calls and supplementary reports into a shared database. While all programs defined overdose reports as incidents where naloxone was administered (e.g., Langabeer et al., 2021), one HEROES article also defined overdose reports as incidents which indicated risky substance use per the responder’s discretion (Yatsco et al., 2020b). HEROES and RIMO programs had structured study criteria, where participants were screened for eligibility, including no current treatment enrollment, regular use of opioids (i.e., using 13+ days in past 30 days), and age 18 years or older. For study purposes, these programs also required participant informed consent before study participation. The Tempe First-Responder ORP did not document informed consent procedures.

Tempe First-Responder ORP also mentioned contacting family members of a person who experienced an overdose to offer support services. Detailed information was not provided on outreach method, context, or content. Data was not collected on the number of family members contacted, their relationship with the person who overdosed, or their sociodemographic characteristics.

Programs conducted outreach 24 hours to 72 hours (White et al., 2021), within one week (Langabeer et al., 2020; Langabeer et al., 2021; Yatsco et al., 2020b), and up to one month (Scott et al., 2020) after an overdose event. HEROES staff who conducted outreach were often a one- or two-person team comprised of a first responder (law enforcement, EMS) and behavioral health staff (peer recovery coach, patient navigator, clinician). RIMO and the Tempe First-Responder ORP documented a behavioral health worker solely conducting post-overdose outreach. The outreach personnel often received special training, such as OUD and motivational interviewing education. The first outreach attempt was typically conducted directly to the residence of the person who had overdosed, where outreach workers would engage the individual. RIMO used a phone call as the first point of outreach, and then conducted in-person outreach to a residence.

### Program size and participant characteristics

Participants are people who were referred to or received services from a given program (detailed in Table 1). Program size ranged from 24 to 81 participants over 8 to 12 months. Programs primarily served men (55.9% - 75%), people of White race (9.1% - 87.5%), and people of Black or African American race (14.6% - 66.7%). Average participant age ranged from 31.6 to 38.2 years (Yatsco et al., 2020b; White et al., 2021; Langabeer et al., 2020; Scott et al., 2020). HEROES and the Tempe-First Responder ORP presented data on homelessness and found a range of individuals reported homelessness or living in temporary housing (27.8% - 76.5%) (Langabeer et al., 2020; White et al., 2021). One HEROES article specifically mentioned omitting individuals who did not report a valid physical address (e.g., street intersections) due to the challenges that this created for recontact (Langabeer et al., 2021). One HEROES article found most participants reported no health insurance (79.4%) (Langabeer et al., 2020). Other studies did not report this information.

### Services offered

All programs offered a variety of services. Every program detailed referral to addiction treatment or assistance with finding addiction treatment as a service offered. All programs provided referral assistance to MOUD. The Tempe First-Responder ORP also mentioned referral to other services, including abstinence-based and detoxification programs. RIMO offered participants MOUD treatment or detoxification program. HEROES exclusively offered rapid MOUD (buprenorphine) induction and subsequent referral to long-term outpatient MOUD services.

After initial participant contact, most programs utilized long-term peer recovery support, although the capacity for peer support varied by program. The Tempe First-Responder ORP behavioral health navigator maintained contact with participants for the 45-day period. HEROES research staff maintained daily participant check-ins for the 90-day study period. The RIMO intervention group received continuous check-ins with a linkage manager to discuss treatment barriers and progress over the study’s 30-day period.

Many programs offered naloxone for those who experienced an overdose. All programs connected participants to wraparound services, such as employment, transportation, food, housing, and health insurance. One HEROES article mentioned connecting participants to mental health treatment services (Yatsco et al., 2020b). The Tempe First-Responder ORP was the only program to mention offering family support and child services.

### Available outcomes of post-overdose interventions

These reported outcomes of post-overdose interventions represent a variety of differing evaluation and research designs, measures, and results. Studies used cross-sectional or longitudinal data (see Table 1 for description of design from each article).

### Evaluation design

All program articles were published by an outside evaluator or researcher, in coordination with or separate from program implementers. The evaluator was most often employed at a university and less frequently at a healthcare system. Studies collected quantitative data, commonly from administrative data collected at baseline and/or after the study period. All of the studies also reported qualitative data, elicited through focus groups, interviews, and/or observation of key partner meetings. In the RIMO study, interviews were conducted with 9 (53%) intervention group participants after the 30-day intervention period. In the remaining studies, qualitative information was derived from key partner focus groups or observations. RIMO was the only study to randomize participants into a treatment or control group.

### Process measures and results

Key partners provided insights into post-overdose intervention implementation. Of these insights, barriers included: stigma within organizations and within the community, lack of leadership buy-in or funding, issues with data sharing among organizations, and structural barriers to participant treatment engagement. Additionally, the HEROES program mentioned the slow growth of a new program coupled with potential participant mistrust of law enforcement involvement as a barrier to intervention utilization (Yatsco et al., 2020b). Key partner reported facilitators were partnerships, communication, understanding of limitations and norms, information sharing, continuous meetings, and leadership buy-in across agencies. Programs also noted different staff roles provided strengths to connect individuals with treatment, for example the use of boundary spanners (i.e., an individual with experience in public safety and public health) to facilitate communication (Langabeer et al., 2021).

Key partners shared anticipated benefits included providing treatment services to a financially vulnerable and treatment hesitant population. An additional benefit of a post-overdose intervention was to supply law enforcement officers with the tools (i.e., naloxone, referrals to other treatment services) and training to save lives (i.e., naloxone training) (White et al., 2021). From the participant interviews, persistent, caring follow-up was a reported facilitator of program operation.

### Outcome measures and results

All programs detailed participant engagement with treatment after post-overdose referral. Of those programs, there was variation in rate of treatment initiation after first contact, ranging from 23% to 81% of participants entering treatment after referral (i.e., participants accepted referral from staff and engaged with treatment at least once) (Yatsco et al., 2020b; White et al., 2021; Langabeer et al., 2020; Scott et al., 2020). HEROES and RIMO detailed MOUD treatment retention after post-overdose treatment referral (i.e., participants accepted referral, engaged with treatment over period defined by program such as 30 days or 90 days). RIMO reported that 44% were retained in MOUD treatment 30-days after first treatment entry. HEROES reported that the MOUD treatment retention rate was 88% at 30-days and 56% at 90-days after first treatment entry (Langabeer et al., 2020). In HEROES, three participants reported return to opioid use but continued to stay engaged with treatment for the 90-day period (Langabeer et al., 2020). The papers reported that there were no deaths or overdose events within the 30- or 90-day periods.

RIMO found the group which received social services referral, individualized treatment counseling and scheduling, and continuous check-in, was significantly more likely to initiate treatment for OUD (intervention 81% vs. control 35%), especially MOUD (81% vs. 18%), and stay engaged in MOUD after the 30-day intervention period (44% vs. 6%), as compared to the control group.

## Discussion

Law enforcement-partnered community-based post-overdose interventions are being implemented around the country with limited knowledge of effectiveness. This scoping review addressed current knowledge on these programs through review of empirical literature. Data on community-based post-overdose intervention context, method of outreach, and evaluation process can provide guidance for implementation of future interventions.

### Preliminary results

#### Use of multidisciplinary teams

Multidisciplinary teams are necessary to address the opioid epidemic (Goodison et al., 2019). Post-overdose intervention programs are another possible space for the use of multidisciplinary teams. Other public health and public safety initiatives have found facilitators for program implementation include buy-in for helping people with addiction, a network of multidisciplinary community partners, and the ability for partners to communicate and share data effectively across sectors (Yatsco et al., 2020a). In the present study, examples of these communications included weekly meetings, triage meetings for individual overdose cases, and having a boundary spanner involved in program implementation. These findings illustrate a salient component to bridging relationships between public safety and public health partners in collaborative programs and can be useful guidance for future program implementers.

#### MOUD engagement and retention

All programs in the current review offered participant access to MOUD. A few programs also referenced referral to detoxification programs. MOUD treatment reduces overdose risk and other poor health outcomes in contrast to detoxification (Wakeman et al., 2020). Other community-based post-overdose interventions have also documented that referral to detoxification or other abstinence-based programs is common among law enforcement partnered programs (Formica et al., 2018; Formica et al., 2021). Findings underscore the need to assess knowledge and beliefs about evidence-based care among participants and also among outreach teams.

Additionally, in the RIMO intervention condition that included MOUD treatment, social services, and counseling (compared to receipt of an informational handout), the MOUD retention rates were significantly higher (Scott et al., 2020). This finding suggests there is added value in providing comprehensive wrap around services along with MOUD treatment. With more rigorous study design, the outcomes of post-overdose interventions could be compared to outcomes yielded by other interventions that aim to retain vulnerable populations in treatment. Future findings can inform whether community-based post-overdose interventions can uniquely communicate with and motivate vulnerable individuals who otherwise may not have entered treatment (Harrison et al., 2021; White et al., 2021).

### Areas for improvement

#### Capacity for services

Individuals with OUD have significantly higher rates of mental illness (Novak et al., 2019). Those with co-occurring mental illness have increased risk for fatal overdose (Webster, 2017) and this risk may have been exacerbated by the COVID-19 pandemic (Cales et al., 2021). The programs in the review did not describe connection to long-term mental health support for individuals. In consideration of high rates of mental illness among this population, this is a gap in service provision which should be explored by future programs to establish whether post-overdose interventions can improve access for mental health services.

Additionally, it is important to consider the capacity and preferences of law enforcement officers to conduct this type of work. Programs in the present review did not discuss the management or alleviation of law enforcement burnout and stress and how this may impact the capacity for law enforcement partners to appropriately engage in post-overdose interventions. To appropriately support and staff post-overdose interventions, it is essential to provide adequate training and resources, and evaluation of how to best utilize law enforcement (Hofer, 2021; Wood, Watson, & Fulambarker, 2017).

#### Participant population

The programs in the current review lacked rural representation. Transportation access and stigma are barriers to substance use treatment in rural areas (Ellis et al., 2021; Haffajee et al., 2019; Kiang et al., 2021; Nguyen et al., 2019). Like findings in the current review, commonly cited barriers to substance use treatment include lack of health insurance and homelessness (Park-Lee, Lipari, & Hedden, 2017). Future rural programs could be tailored to these needs, including medical staff education to reduce stigma (Volkow, 2020b) and establishment of a robust referral and service navigation effort to increase access to health insurance and stable housing.

HEROES implementers noted distrust in law enforcement was a potential area for lack of program engagement (Yatsco et al., 2020b). Distrust in public institutions is more common among people who have been historically and are presently marginalized, including Black, Latinx, and Indigenous communities. This historic and present marginalization is caused and compounded by structural racism (Boyd et al., 2020). Structural racism is also heavily linked to criminal justice involvement and substance use among this population (FXB Center for Health & Human Rights at Harvard University, 2020). When designing and implementing post-overdose interventions it is critical to equitably reach all members of a given community, prioritize reaching those who are at highest risk for overdose, and to divert people from the criminal justice system into evidence-based systems of care (FXB Center for Health & Human Rights at Harvard University, 2020).

In addition, programs in the current review predominantly served White males, further demonstrating the need for interventions tailored to women and minoritized populations. Researchers have proposed that public health workers can bridge this gap of law enforcement distrust through building relationships with community members and disseminating evidence-based education about public health initiatives, to promote health-supporting programs such as post-overdose interventions (Fleming et al., 2021). Further, staff training needs to consider how practices such as warrant checking prior to outreach may be experienced as traumatizing by prospective participants, delay outreach efforts, and function as a barrier to care that disproportionately affects justice-involved individuals (Tori et al., 2022).

#### Facilitating services for other individuals

In the present review, outreach and support service provision for family members following a loved one’s fatal or non-fatal overdose were not described. Witnessing an overdose and experiencing grief can require psychological care (American Psychiatric Association, 2013; Feigelman et al., 2011). Individuals who experience overdose-related trauma or grief often do not seek services due to stigma (Bergman, Axberg, & Hanson, 2017). By conducting outreach to these individuals, post-overdose interventions are filling a healthcare service gap. Future efforts for post-overdose interventions could benefit from developing, implementing, and evaluating program components which serve others affected by opioid overdose. For example, provision and evaluation of training materials for specialized law enforcement partners on how to best serve an overdose witness.

Support services for children who were involved with an overdose were not well-documented in the current study. Including services for children within a post-overdose intervention could be a useful avenue to increase public health (Bergman et al., 2017). One complexity of intervening with children after their exposure to a drug overdose is the risk of child removal for perceived safety risk (Thumath et al., 2021). Child removal is associated with increased odds of parental overdose (Thumath et al., 2021) and unmet healthcare needs (Canfield et al., 2017; Doab et al., 2015). Post-overdose interventions could benefit from connecting individuals with integrated services including peer recovery coach support, child-welfare worker, and MOUD treatment referral (Hall et al., 2015; Hall et al., 2016).

#### Need for rigorous evaluation of implementation and outcomes

The current state of post-overdose intervention knowledge is limited and efficacy has not been established. Robust implementation science studies utilize mixed-methods design to further expand knowledge on factors which challenge and facilitate early implementation of novel programs (Powell et al., 2013). To evaluate public health program outcomes, other study methodologies include collection of administrative data (i.e., from first responders, criminal justice facilities) to track long-term outcomes (Bigelow et al., 2021) and collection of participant self-reported data. Outcomes to measure from these data sources include use of MOUD, overdose, hospitalization, mortality, arrest, and incarceration. Collecting data points through these sources will provide additional triangulation of findings.

### Strengths & Limitations

This scoping review defined, synthesized, and discussed the evidence base for community-based post-opioid overdose interventions. A limitation of this scoping review was the inability to assess articles published after the search date. Post-overdose interventions are emergent in public health literature and it is expected that articles will continue to be published on the topic. The present review did not evaluate articles published in a non-English language or outside of the United States to capture interventions most relevant to the culture and current state of the United States’ opioid epidemic. Publications outside of these criteria could provide additional insight to post-overdose interventions. Another limitation of the present review is that programs only documented in gray literature (i.e., documented in government reports) were excluded from the present search to capture only evidence-based program outcomes. The present review does not capture the potential diversity and variation of gray literature-documented post-overdose interventions. Programs included in this review represent few evaluated models and, therefore, may have limited generalizability to broader implementation and effectiveness of post-overdose interventions operating across the country. Previous post-overdose intervention reviews found programs lacked robust outcome data to suggest program effectiveness because programs were emergency responses to combat the opioid epidemic (Bagley et al., 2019). Further evaluation needs to be built upon more robust study designs to substantiate findings on community-based post-overdose interventions.

## Conclusion

This scoping review found that the current knowledge base on community-based post-overdose interventions is emergent and requires further research. Post-overdose teams are multidisciplinary, comprised of law enforcement, public health, healthcare, and others, and aim to refer individuals who experience an opioid overdose to evidence-based treatment and wrap-around services. To work towards this goal, future programs can take guidance from facilitators documented in the peer-reviewed literature, including key partner collaboration and understanding across sectors, and the ability for key partners to communicate with participants to motivate and support recovery engagement. Challenges to program success were also documented in the literature including inability to contact hard-to-reach individuals and structural barriers to treatment engagement. In this scoping review, some gaps in service provision were identified including lack of rural programs, mental health support, and support for family affected by overdose. Future interventions could benefit from utilization of public health workers to reduce barriers for minoritized populations who may be mistrustful of law enforcement. To facilitate the growth of these innovative programs, more rigorous mixed-method evaluation of community-based post-overdose interventions are needed to establish a robust program model for future implementers.

## Supplementary Information

**Additional file 1.** Appendix A: PRISMA checklist for scoping reviews. Appendix B: Search terms.

## Data Availability

The datasets used and/or analyzed during the current study are available from the corresponding author on reasonable request.

## Abbreviations

OUD: opioid use disorder
MOUD: medication for opioid use disorder.
