# Supplementary material for: A scoping review of community-based post-opioid overdose intervention programs: implications of program structure and outcomes
Source: Health Justice. 2023 Jan 28;11:3. doi: 10.1186/s40352-022-00201-w (PMC9883127; doi:10.1186/s40352-022-00201-w)
Supplement: Supplementary file 1 — Additional file 1. Appendix A: PRISMA checklist for scoping reviews. Appendix B: Search terms. [file 40352_2022_201_MOESM1_ESM.docx]

**Appendix A: PRISMA Checklist for Scoping Reviews**

|  | Status |
| --- | --- |
| **Title** |  |
| Title | Completed |
| **Abstract** |  |
| Structured summary | Completed |
| **Introduction** |  |
| Rationale | Completed |
| Objectives | Completed |
| **Methods** |  |
| Protocol registration | N/A |
| Eligibility | Completed |
| Information sources | Completed |
| Search | Completed |
| Selection of sources of evidence | Completed |
| Data charting process | Completed |
| Data items | Completed |
| Critical appraisal of individual sources of evidence | Completed |
| Synthesis of results | Completed |
| **Results** |  |
| Selection of sources of evidence | Completed |
| Characteristics of sources of evidence | Completed |
| Critical appraisal within sources of evidence | Completed |
| Results of individual sources of evidence | Completed |
| Synthesis of results | Completed |
| **Discussion** |  |
| Summary of evidence | Completed |
| Limitations | Completed |
| Conclusions | Completed |
| **Funding** |  |
| Funding | Completed |

**Appendix B: Search terms**

| Database | Search terms |  |
| --- | --- | --- |
| PubMed  and  Web of Science | ("Post-overdose"[All Fields] OR ("Post"[All Fields] AND "opioid overdose"[All Fields]) OR ("Post"[All Fields] AND ("opiate overdose"[MeSH Terms] OR ("opiate"[All Fields] AND "overdose"[All Fields]) OR "opiate overdose"[All Fields] OR ("opioid"[All Fields] AND "overdose"[All Fields]) OR "opioid overdose"[All Fields])) OR ("After"[All Fields] AND ("drug overdose"[MeSH Terms] OR ("drug"[All Fields] AND "overdose"[All Fields]) OR "drug overdose"[All Fields] OR "overdose"[All Fields] OR "overdoses"[All Fields] OR "overdosed"[All Fields] OR "overdosing"[All Fields])) OR ("Post"[All Fields] AND ("drug overdose"[MeSH Terms] OR ("drug"[All Fields] AND "overdose"[All Fields]) OR "drug overdose"[All Fields] OR "overdose"[All Fields] OR "overdoses"[All Fields] OR "overdosed"[All Fields] OR "overdosing"[All Fields])))  AND  ("intervention s"[All Fields] OR "interventions"[All Fields] OR "interventive"[All Fields] OR "methods"[MeSH Terms] OR "methods"[All Fields] OR "intervention"[All Fields] OR "interventional"[All Fields] OR ("outreach"[All Fields] OR "outreaches"[All Fields] OR "outreaching"[All Fields]) OR (("assert"[All Fields] OR "asserted"[All Fields] OR "asserting"[All Fields] OR "assertion"[All Fields] OR "assertions"[All Fields] OR "assertive"[All Fields] OR "assertively"[All Fields] OR "assertiveness"[MeSH Terms] OR "assertiveness"[All Fields] OR "assertives"[All Fields] OR "asserts"[All Fields]) AND ("outreach"[All Fields] OR "outreaches"[All Fields] OR "outreaching"[All Fields])) OR (("critical"[All Fields] OR "critically"[All Fields]) AND ("time"[MeSH Terms] OR "time"[All Fields]) AND ("intervention s"[All Fields] OR "interventions"[All Fields] OR "interventive"[All Fields] OR "methods"[MeSH Terms] OR "methods"[All Fields] OR "intervention"[All Fields] OR "interventional"[All Fields])) OR (("genetic linkage"[MeSH Terms] OR ("genetic"[All Fields] AND "linkage"[All Fields]) OR "genetic linkage"[All Fields] OR "linkage"[All Fields] OR "linkages"[All Fields]) AND ("connect"[All Fields] OR "connectable"[All Fields] OR "connected"[All Fields] OR "connecting"[All Fields] OR "connection"[All Fields] OR "connectional"[All Fields] OR "connections"[All Fields] OR "connective"[All Fields] OR "connectives"[All Fields] OR "connectivities"[All Fields] OR "connectivity"[All Fields] OR "connects"[All Fields] OR "connexion"[All Fields] OR "connexions"[All Fields])) OR (("communal"[All Fields] OR "communalism"[All Fields] OR "communalities"[All Fields] OR "communality"[All Fields] OR "communally"[All Fields] OR "commune"[All Fields] OR "communes"[All Fields] OR "community s"[All Fields] OR "communitys"[All Fields] OR "residence characteristics"[MeSH Terms] OR ("residence"[All Fields] AND "characteristics"[All Fields]) OR "residence characteristics"[All Fields] OR "communities"[All Fields] OR "community"[All Fields]) AND ("navigability"[All Fields] OR "navigable"[All Fields] OR "navigate"[All Fields] OR "navigated"[All Fields] OR "navigates"[All Fields] OR "navigating"[All Fields] OR "navigation"[All Fields] OR "navigational"[All Fields] OR "navigations"[All Fields] OR "navigator"[All Fields] OR "navigator s"[All Fields] OR "navigators"[All Fields])) OR "follow-up"[All Fields] OR (("follow"[All Fields] OR "followed"[All Fields] OR "following"[All Fields] OR "followings"[All Fields] OR "follows"[All Fields]) AND "up"[All Fields]))  AND  ("communal"[All Fields] OR "communalism"[All Fields] OR "communalities"[All Fields] OR "communality"[All Fields] OR "communally"[All Fields] OR "commune"[All Fields] OR "communes"[All Fields] OR "community s"[All Fields] OR "communitys"[All Fields] OR "residence characteristics"[MeSH Terms] OR ("residence"[All Fields] AND "characteristics"[All Fields]) OR "residence characteristics"[All Fields] OR "communities"[All Fields] OR "community"[All Fields] OR (("communal"[All Fields] OR "communalism"[All Fields] OR "communalities"[All Fields] OR "communality"[All Fields] OR "communally"[All Fields] OR "commune"[All Fields] OR "communes"[All Fields] OR "community s"[All Fields] OR "communitys"[All Fields] OR "residence characteristics"[MeSH Terms] OR ("residence"[All Fields] AND "characteristics"[All Fields]) OR "residence characteristics"[All Fields] OR "communities"[All Fields] OR "community"[All Fields]) AND ("based"[All Fields] OR "basing"[All Fields])) OR "community-based"[All Fields]) |  |
| PsycInfo | ("post overdose" OR "drug overdose" OR "opioid overdose" OR "post opioid overdose" OR "after overdose") AND ("intervention" OR "program" OR "outreach" OR "assertive outreach" OR "critical time intervention" OR "follow up" OR "service") AND ("community based" OR "community-based" OR "community program" OR "community navigation" OR "community") |  |
